# Physical and mental load of tactical athletes during Arctic military training

**DOI:** 10.3389/fspor.2026.1861009

**Published:** 2026-07-14

**Authors:** N. H. van den Berg, X. Michaud, J. P. Vaara, T. Ojanen, H. Kyröläinen, A. Martin, F. Haman, R. Heikkinen, J. Van Cutsem, N. Pattyn, J. Helen, G. Simonelli

**Affiliations:** 1 Department of Psychology, Faculty of Arts, University of New Brunswick, Fredericton, NB, Canada; 2 VIPER Research Unit, LIFE Department, Royal Military Academy, Brussels, Belgium; 3 Centre d'études avancées en médecine du sommeil (CEAMS), Centre Intégré Universitaire Santé Services Sociaux Nord-de-Île-de-Montréal (CIUSSS-NÎM), Montreal, QC, Canada; 4 Department of Psychology, Faculty of Arts and Sciences, Université de Montréal, Montreal, QC, Canada; 5 Department of Leadership and Military Pedagogy, National Defence University, Helsinki, Finland; 6 Faculty of Sport and Health Sciences, University of Jyväskylä, Jyväskylä, Finland; 7 Finnish Defence Research Agency, Finnish Defence Forces, Riihimäki, Finland; 8 Technical Unit, Fundación para la Investigación Biomédica del Hospital Gregorio Marañón, Instituto de Investigación Sanitaria Gregorio Marañón (IiSGM), Madrid, Spain; 9 Faculty of Health Sciences, University of Ottawa, Ottawa, ON, Canada; 10 Statistical Analysis Services, Analyysitoimisto Statisti Oy, Jyväskylä, Finland; 11 Human Physiology Department (MFYS), Vrije Universiteit Brussel, Brussels, Belgium; 12 Brain, Body & Cognition, Vrije Universiteit Brussel, Brussels, Belgium; 13 Department of Neuroscience, Faculty of Medicine, Université de Montréal, Montreal, QC, Canada; 14 Department of Medicine, Faculty of Medicine, Université de Montréal, Montreal, QC, Canada

**Keywords:** aerobic performance, illness prevention, military performance, physical fitness, sleep, tactical athletes

## Abstract

**Introduction:**

Tactical athletes require consistent physical and mental exertion to succeed in their mission, often under life-threatening conditions. Soldiers as tactical athletes are exposed to a myriad of stressors that may deteriorate performance, especially in harsh military environments. It remains unclear which combination of traits, habits, and characteristics of soldiers affects the likelihood of performance decrement during military operations.

**Methods:**

This observational cohort study investigated the differences between a physical-load group and a mental-load group of cadets (*n* = 49) during a 2-week near-Arctic winter training. One group comprised first-year cadets who had physical demands, while the second-year cadets had more leadership requirements (command and control) and fewer physical demands. The chance occurrence of a viral infection in the first-year cadets provided a naturalistic experiment to study vulnerability to the illness. Demographics, trait resilience, exercise training behavior, and sleep habits were assessed prior to training. Throughout training, wrist-worn accelerometers monitored sleep-wake activity, and cadets completed daily self-report entries on perceived sleepiness, stress, and mood states. Body composition was measured before and after training.

**Results:**

Findings showed the following: (1) poorer self-reported mood, stress, and sleepiness during preparation and cleanup phases than during simulated combat among the mental-load group, but for the physical-load group, these measures were poorest during the simulated combat; (2) an overall decrease in fat mass after the training; and (3) survival analysis demonstrated that older age and less frequent endurance training habits significantly predicted the likelihood of illness during training.

**Discussion:**

Leaders of tactical athletes should be aware of the taxing mental load not just for the main event itself, but for preparation and cleanup phases. Military experience and aerobic fitness might attenuate the likelihood of succumbing to viral illnesses during missions involving close quarters.

## Introduction

For more than a decade, military medicine has expanded research from a solely curative approach to preventive interventions that enhance health and well-being (1–3). These preventive interventions aim to enhance retention of personnel, expand the existing duty of care, and optimize performance. In the United States, such a framework is termed the “Performance Triad” and is structured around the World Health Organization's “pillars of health”, which include physical activity, sleep, and nutrition (3). This framework summarizes the interdependency of performance, sleep, mood, and stress during military operations.

Depletion of both physical and psychological resources can compromise the mission, and military operations test the physical and mental fortitude of soldiers (4–6). Soldiers have accordingly been deemed tactical athletes (7), in line with the high physical and mental demands they experience, often in life-threatening and unpredictable environments (8, 9). The goal for tactical athletes is to accomplish a mission, often by pushing the boundaries of mental and physical performance while maintaining overall safety and recovery. Most of the physical and mental depletions observed in tactical athletes are replicated during training exercises, allowing for experimental observation studies akin to real-world scenarios.

Theoretical models (10–13) suggest that, whereas some factors which contribute to tactical athletes' depletion of resources are constraints of the environment and therefore cannot be modified, other factors can be modified in anticipation of the mission. More specifically, traits or habits that exist even prior to a mission might prevent performance decrements. The aim of the present study is to identify, in an intensive near-arctic winter military training, the physiological and psychological determinants of tactical athlete (i.e., soldier) performance.

However, performance is a multimodal construct for which there is no gold standard of measurement. Hence, modeling performance usually involves, at best, a reductionist approach depending on *a priori* assumptions or, at worst, cherry-picking from a wealth of recorded data for statistically significant findings. This work uses the existing framework of the “pillars of health” to measure physical activity, sleep, and mood in soldiers. In the present study, we investigated physical and mental load as framed through these pillars of health—specifically fitness habits, sleep habits, and psychological trait resilience—both as preconditions of the training and as situational variables during the training.

Regarding physical activity, aerobic fitness is among the most recognized factors to mitigate performance decrements among military personnel (14–17). It is inversely associated with perceived stress during survival training (17), and it is the strongest predictor of dropout rates from military training (18). Anaerobic fitness might also improve stress tolerance to prevent fatigue (4, 19) and thus can also be considered in preparation for a mission.

Poor sleep, whether in quantitative or qualitative terms, is also a major cause of performance decrement. Deficient sleep is pervasive in military forces, where soldiers report higher rates of subjective sleepiness and shorter sleep durations compared to the general population (11, 20). However, alterations in both sleep quantity and quality might arise from non- modifiable operational mission demands, and thus solutions based on “sleep more” or “sleep better” are rarely an option (11). Instead, the distal health outcomes of poor sleep behavior during missions, for example, fatigue, can be explored.

Trait resilience is also a likely predictor of tactical athlete performance. Trait psychological resilience or hardiness is the ability to endure hardships with minimal compromise to mission success or individual health (16). Resilience is shown to moderate the effect of physical and mental fatigue experienced from combat (21), even after controlling for nutrition and physical fitness (22), and predicts success in Special Forces selection (23, 24).

Aside from the pillars of health that predict success, tactical athletes also face threats to their performance. Among these threats, stress is widely associated with impaired immune systems (25). The heavy physical demands, poor sleep behavior, and threats to trait resilience that tactical athletes endure during their duties can therefore manifest through illness (26–29). Often, these physiological impairments are coupled with psychological effects, and in soldiers, this is usually manifested through detrimental effects on mood (30, 31) and higher perceived stress (32).

Applied research during military training or deployment requires a continuous balance between hypothesis-driven research designs in complex multimodal constructs on the one hand, and opportunities for measurements as dictated by operational constraints on the other hand, while minimizing hazards of the environment. The present study is no exception. Whereas we aimed to investigate the difference in the factors cited above in two groups undergoing winter military training—one undergoing more physical, executant, tactical level training versus another one undergoing more mental, executive, operational level training—the spread of a viral infection amongst one of our groups (the physical training group) created a naturalistic experiment, allowing us to investigate illness prevalence according to our existing variables.

In the present study, we sought to 1) identify traits and behaviors that impact fatigue during a military training exercise and 2) identify how detriments to the physical and mental readiness of tactical athletes manifest. We first hypothesized that poor trait resilience, exercise training behavior, and sleep restriction would impair mood and physiological outcomes. After observing the illness, we then sought to (3) identify the factors that explained those who succumbed to the viral infection as measured by medical leave from training.

## Materials and methods

### Participants

The present study observed first-year and second-year volunteer cadets participating in a winter military training. Cadets provided written informed consent to participate in this study, and ethical approval was granted by the National Defence University (AS29340). Participation was optional, and a chain of command was not present during recruitment to prevent coercion. In total, *n* = 49 cadets opted in. All data collection and management were performed according to the Declaration of Helsinki and are GDPR compliant. As these military personnel are screened for medical conditions precluding exclusion from active duty, we did not deem it necessary to specify additional exclusion criteria.

The demands of the training differed according to the year of experience for cadets: first-year cadets slept in tents and had more physically demanding requirements (e.g., simulated combat, ski marches, live fire exercises), thus practicing tactical skills. By contrast, second-year cadets slept in barracks, had a more commanding role during the training, planned the training schedule, and trained the first-year cadets on live fire exercises, hence practicing their operational command and control skills. The original aim was to investigate the differences in sleep, mood, physical activity, and body composition in these two groups.

Incidentally, a viral infection spread throughout first-year cadets during training, as noted by observations of the investigators and daily self-report diaries. In total, 11 (29.6%) first-year cadets reported flu-like symptoms and were granted medical leave, dispensing them from several days of training. Rather than excluding these participants, we used this occurrence as a naturalistic experiment regarding susceptibility to illness, considering the relevance of this occurrence for our initial research question. We thus maintained the inclusion of these cadets by assigning them to a third group. In this way, our study examines three groups: first-year cadets who did not report symptoms of flu/illness (HEALTH1; *n* = 16), first-year cadets who reported symptoms of flu/illness (FLU1; *n* = 11), and second-year cadets, none of whom reported symptoms of flu/illness (HEALTH2; *n* = 22).

### Study design: performance determinants during winter training

This observational cohort study examined determinants of performance related to physical activity, sleep, body composition, and mood (as further detailed in the following section) in cadets in the framework of a 13-day winter training.

The first five days of training comprised tactical combat exercises (including live fire) and strenuous ski marches. On the 6th day/7th night, first-year cadets engaged in a 72-hour simulated combat exercise. Cadets were given a small extended prophylactic sleep opportunity the day before this simulated sustained operations combat period, and they were given an extended recovery sleep opportunity afterwards. Accordingly, we structured our analysis around four phases of the winter training, divided as follows: a “Standard” 72-hour period during training, featuring typical 6-hour sleep opportunities each night; a 10-hour “Prophylactic” overnight sleep opportunity in anticipation of simulated combat; the 72-hour combat simulation (“Combat”) with restricted sleep opportunity; and a 30-hour “Recovery” phase following Combat. None of the FLU1 cadets participated in the Combat phase. This condition/time variable and the grouping variables (HEALTH1, FLU1, and HEALTH2) define the independent variables of our design.

**Figure 1:**
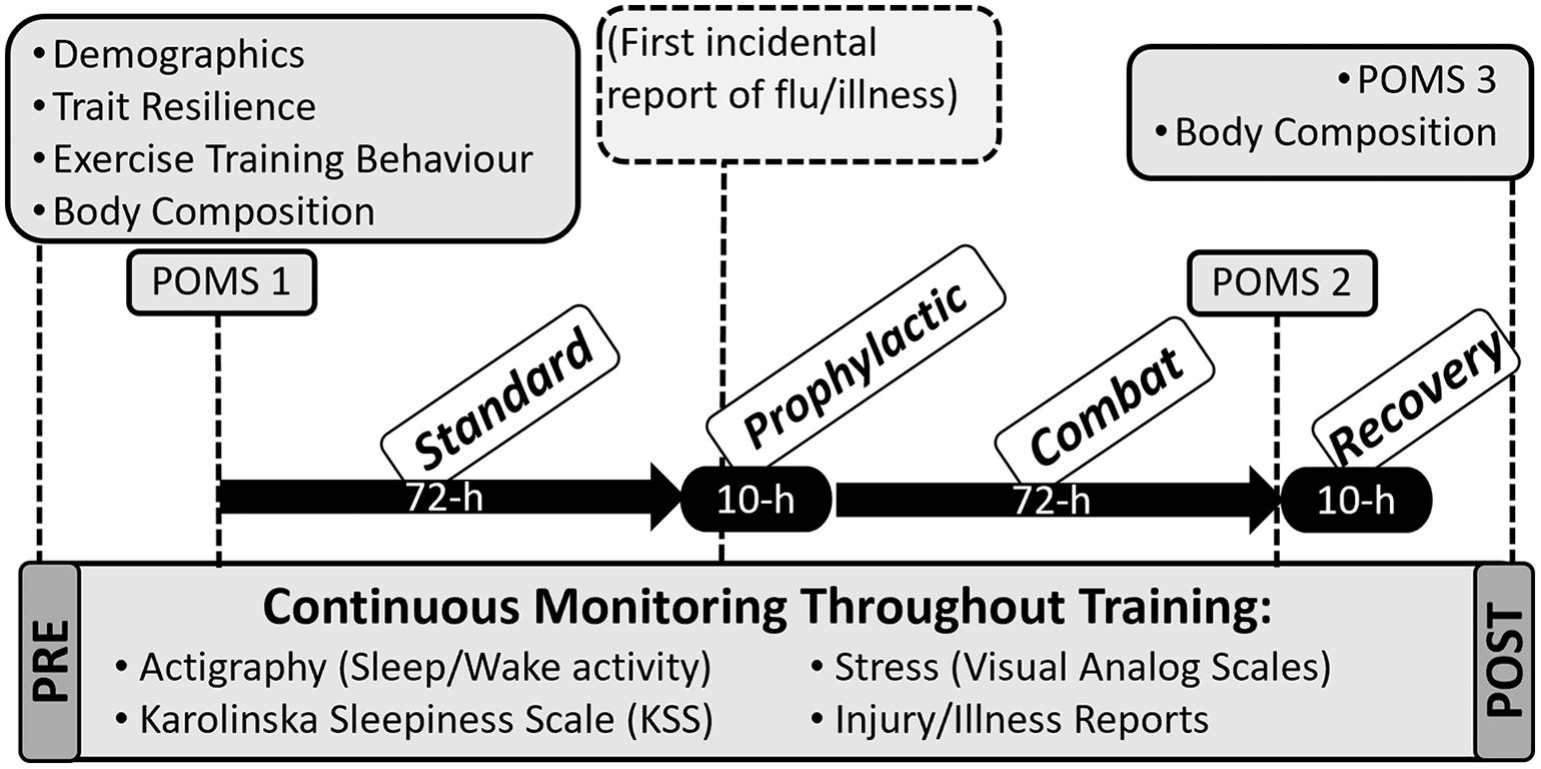
Overview of winter training as it relates to measures collected during its relevant phases of the training: standard, prophylactic, combat, and recovery. Demographics, trait resilience, and exercise training behavior were acquired prior to winter training. Body composition was collected before and after the winter training. In addition to continuous monitoring of several variables throughout winter training, Profile of Mood States (POMS) assessments were collected at three timepoints. The first incidental report of flu/illness occurred after the Standard Phase.

The dependent variables are described in the following section. Figure 1 summarizes our study design with the obtained measurements according to the different time points.

### Measurements

#### Demographics, military experience, and trait resilience

Prior to the start of training, participants completed a questionnaire including age and months of military service. Participants also completed the 25-item Connor-Davidson Resilience Questionnaire (33). All 25 items were summed for a global score of trait psychological resilience.

#### Sleep-wake activity

Sleep-wake activity was recorded through wrist-worn accelerometry “Actiwatch” (Actiwatch Spectrum Plus; Phillips/Respironics, Murrysville, USA), with a sampling rate of 32 Hz, in 30-second epochs. The Actiwatch records the integration of movement and direction of movement as activity counts. If the activity count was less than the wake threshold value, it was scored as sleep. Wake threshold value was set to a medium threshold of 40. Participants wore the Actiwatch on their non-dominant hand; *n* = 2 participants were left-handed. Computed outcome variables were total sleep time and daily activity count during wake time (i.e., physical load).

#### Prior exercise training behavior

Self-report questions assessed how many times in a week, on average, cadets engage in “endurance-type exercise” or “strength-type exercise”. Responses to both questions were scaled from 0 to 7, with higher scores indicating how often they engage in the exercise per week.

#### Body composition

Body composition was measured once before and once after winter training using multi-frequency bioelectrical impedance analysis (BIA; InBody, 770 Biospace Co., Ltd., Seoul, South Korea) after an overnight fast. Measurements were standardized, comprising a consistent time of day, fasting status, and clothing. Total body and segmental muscle and fat mass were calculated through proprietary algorithms (34), as is often used in applied military research (18, 35, 36).

#### Mood

Participants responded to a 40-item version of the Profile of Mood States (37) questionnaire (POMS) at three timepoints: 3 days prior to winter training, after the Combat phase, and on the last day of winter training. The POMS is a robust assessment of six mood factors: Fatigue-Inertia, Tension-Anxiety, Anger-Hostility, Vigor-Activity, Depression-Dejection, Confusion- Bewilderment, and High Esteem-Low Esteem.

#### Daily self-report

##### Sleepiness

Subjective state sleepiness was measured using the Karolinska Sleepiness Scale (KSS) (38). Through a single Likert scale item, participants rated their state sleepiness from 1 (“Extremely Alert”) to 9 (“Very sleepy, great effort to keep awake, fighting sleep”).

##### Stress

Participants indicated on a horizontal 100-mm line (Visual Analog Scale) their current level of stress, with the left extreme marked as “Not at all stressed” and the right extreme marked as “Very Stressed”. Scores were measured by the distance from the left extreme in mm, with longer distances (i.e., closer to the right extreme) indicating higher levels of stress.

##### Open-ended report

At every daily entry, participants answered (through Yes or No questions) if they sustained injuries or illness, and if yes, were asked to briefly describe the injury/symptoms.

### Statistical analyses

Parametric model assumptions were checked and met unless otherwise specified. Power analysis indicated that, for a two-tailed alpha set to 0.05, and an expected small effect size (*f* = 0.2) when comparing three groups, our sample size of 49 is adequate for achieving statistical power of 0.80; however, the incidental finding of the FLU1 group created unequal sample sizes of 16 (HEALTH1), 11 (FLU1), and 22 (HEALTH2), thus the power analysis is an approximation for the primary analyses of mixed-effects models for this observational study.

We first characterized each group's demographics, traits, and habits prior to the mission (i.e., age, military experience, trait resilience, prior exercise training) and the situational demands during the mission (i.e., TST and physical activity during winter training). The independent variables were group (HEALTH1, HEALTH2, and FLU1) and, in the case of assessing sleep-wake behavior, the training phase (Standard, Prophylactic, Combat, and Recovery). The dependent variables were age, military experience, trait resilience, prior exercise training, TST, and physical activity.

We then analyzed differences between groups in terms of the physical and mental performance outcomes that occurred throughout the winter training. The independent variables remained the same (i.e., the groups and training phases), whereas the dependent variables were body composition, mood, sleepiness, and stress. A survival analysis then determined which factors prior to the mission predicted likelihood of reporting illness. Each separate analysis is described below.

#### Age and military experience

Independent samples *t*-tests compared age and months of service between the HEALTH2 group and all first-year cadets combined (HEALTH1 + FLU1 groups). Subsequent independent samples t-tests compared age and months of service between HEALTH1 and FLU1 groups.

#### Trait psychological resilience

A one-way ANOVA assessed the difference between HEALTH1, FLU1, and HEALTH2 groups on trait resilience. Planned comparison independent t-tests are reported between HEALTH1 and HEALTH2 groups, whereas *post-hoc* t-tests concerning FLU1 are only included if the omnibus *F*-tests were significant for any of these traits or baseline variables.

#### Total sleep time and physical activity

A 4 × 3 Phase by Group repeated measures ANOVA characterized the trajectory of both recovery (i.e., TST) and physical load (i.e., mean daily wake activity counts) across training phases between groups. Importantly, this ANOVA is intended to simply describe the different phases, to confirm that the three groups are indeed distinct, with different sleep-wake activity schedules throughout the training.

Subsequently, planned comparison independent samples t-tests tested for differences between HEALTH1 and FLU1 groups on TST and mean wake activity counts, though only for the phases occurring prior to symptom onset (i.e., at the Standard and Prophylactic phases). Comparisons with HEALTH2 were not performed, given the inherent differences in schedules.

#### Prior exercise training behavior

A one-way ANOVA assessed the difference between HEALTH1, FLU1, and HEALTH2 groups in terms of exercise training habits prior to the winter training, specifically engagement in aerobic training and anaerobic (strength) training. Planned comparison independent t-tests are reported between HEALTH1 and HEALTH2 groups, whereas *post-hoc* t-tests concerning FLU1 are only included if the omnibus *F*-tests were significant.

#### Body composition

For each participant, we first calculated the change (mean Δ) in total body mass, muscle mass, and fat mass (i.e., kilograms after winter training minus kilograms before winter training) and subsequently performed a one-sample t-test for each of these metrics to assess overall changes in body composition throughout the training. We then compared body composition between HEALTH1, HEALTH2, and FLU1 groups using a one-way ANOVA and planned comparison *t*-tests.

#### Mood

We log-transformed factors of Tension, Anger, Depression, and Confusion from the POMS scale due to positively skewed distributions of raw scores. This allowed us to run a series of 3 × 3 Group (HEALTH1, FLU1, and HEALTH2) by Mood report (Pre-Departure, Post-Combat, and Recovery) linear mixed models with participant entered as random effects, on all seven POMS factors, to identify fluctuations in mood throughout the training and how this differed between groups.

#### Subjective sleepiness

We tested for differences in self-reported sleepiness between the HEALTH1 and FLU1 cadets prior to symptom onset, during the “Standard” phase of winter training, using an independent t-test. Comparisons during other phases were not performed given the bedridden illness during other phases among the FLU1 group, which limited responses from these cadets.

#### Perceived stress

Incomplete self-reported daily stress levels limited data availability to 5 days out of the 10-day training. Also, too few reports from the FLU1 group reported consistently on levels of stress, and the FLU1 group was excluded from this analysis. A 5 × 2 Phase of Training (Training Start, Standard, Prophylactic, Combat, and Recovery) by Group (HEALTH1 and HEALTH2) linear mixed model assessed changes in daily self-reported stress.

#### Likelihood of reporting illness

To determine which traits or habits prior to the training might predict resilience to flu/illness symptoms, we conducted a Cox regression survival analysis using the likelihood ratio test criterion on first-year cadets. We computed Akaike Information Criterion (AIC) difference values to observe the expected predictability of each model. The time factor was the first day of symptom onset for each cadet with flu-like symptoms, entered against censored data for cadets who did not report any symptoms. We determined the order of forward variables first by factors that are non-modifiable or trait-like (i.e., age, trait psychological resilience), followed by factors that are modifiable (i.e., aerobic and strength training). Performance outcomes were not included, given that they occurred *a posteriori*, and could not be a predictor of resilience if the illness had already happened. In this way—for this survival analysis only—the independent variables were age, trait psychological resilience, and prior exercise training, and the dependent variable was the time to the first day of symptom onset.

## Results

### Demographics

The average age of cadets, independent of their group, was *M* = 23 ± 2 (range = 20–29) years, whereas military service duration was *M* = 29 ± 13 months (range = 15–90). As expected, the second-year cadets had more months of service (*M* = 35 ± 7 months, range = 24–48) than the first-year cadets (*M* = 24 ± 15 months, range = 15–90), *t*(46) = 2.99, *p* = 0.004, *d* = 0.85; however, no significant differences in age were observed between second-year (*M* = 22 ± 2 years, range = 20–29) and first-year cadets (*M* = 22 ± 2 years, range = 20–27), *t*(46) = 0.73, *p* = 0.733, *d* = 0.10.

Among first-year cadets, the FLU1 group was significantly older (*M* = 23 ± 3 years, range=20–27) than the HEALTH1 group (*M* = 21 ± 1 years, range = 21–25), *t*(25) = 2.94, *p* = 0.007, *d* = 1.06. The FLU1 group also had longer military service (*M* = 32 ± 2 months, range = 17–90) than the HEALTH1 group (*M* = 19 ± 4 months, range = 15–30), *t*(25) = 2.46, *p* = 0.021, *d* = 0.86.

### Trait resilience

There were no significant differences when comparing trait resilience scores using a one- way ANOVA between HEALTH1 (*M* = 78 ± 11), FLU1 (*M* = 82 ± 19) and HEALTH2 groups (*M* = 78 ± 7), *F*(2,44) = 0.79, *p* = 0.457. *η_p_^2^* = 0.03.

### Total sleep time and physical activity

**Figure 2:**
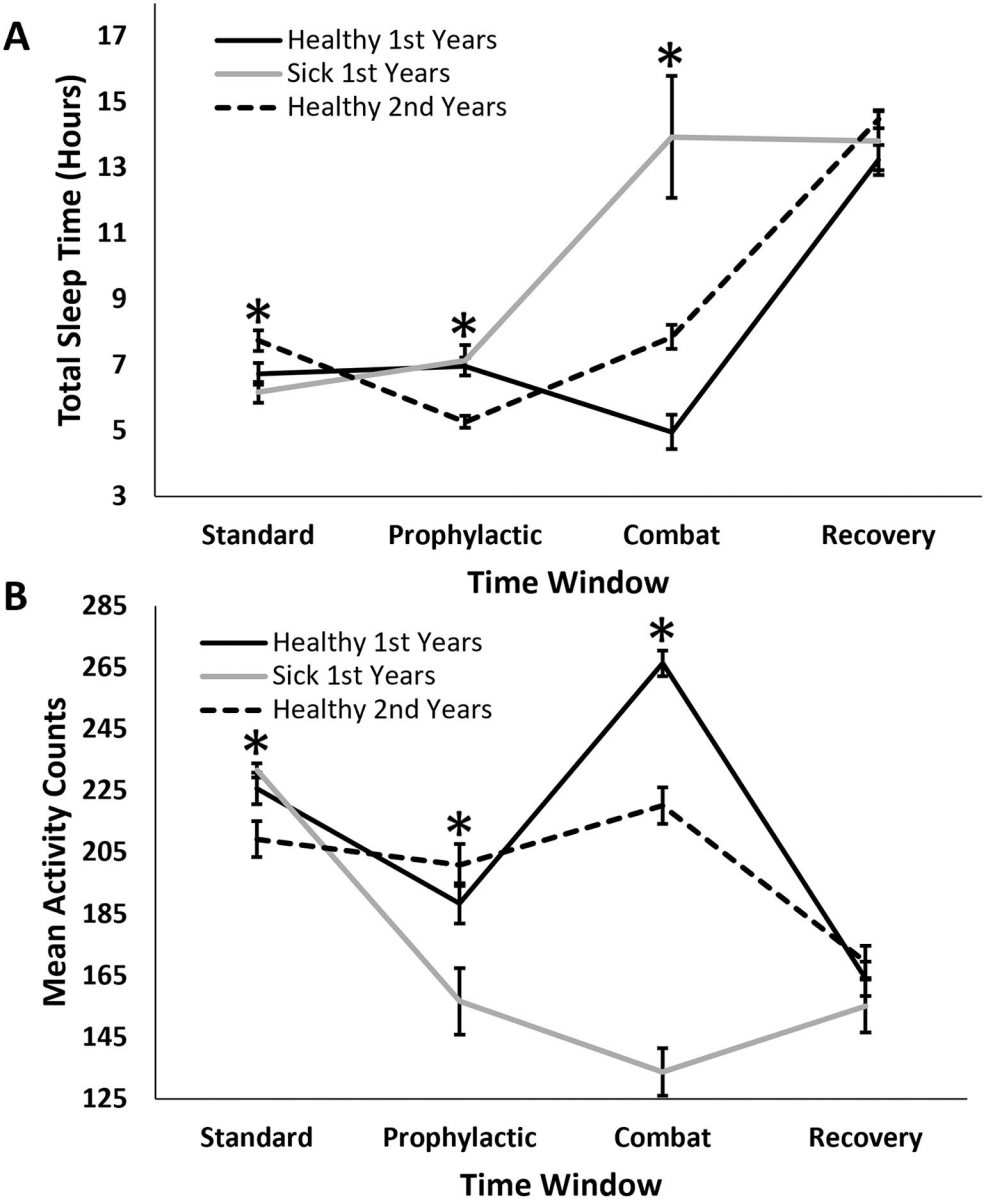
Distinction between groups across phases of training based on wrist-worn actigraphy for total sleep time **(A)** and mean activity counts **(B)**. Significant differences (all *p* < 0.05) reflect different demands and responsibilities depending on the group. During the Standard phase, healthy second years had significantly more total sleep time and significantly less mean activity counts than both healthy and sick first years. The prophylactic phase had less total sleep time (i.e., less prophylactic sleep opportunity) among second years. The sick first years showed the lowest activity counts at the prophylactic and combat phases, complemented by the highest total sleep time during the combat phase (i.e., reflecting bedridden illness/non-participation). Meanwhile, healthy first years showed the lowest total sleep time and highest mean activity counts, thus reflecting the heaviest amount of physical demand, especially during the combat phase. All groups took advantage of the recovery opportunity, reflected by high total sleep time and low mean activity count. These patterns of sleep and activity illustrate the justification for group comparisons, given the inherently different schedules and roles depending on the group. * indicates group differences with *p* < 0.05.

Figure 2 summarizes the findings for both total sleep time and mean activity counts during wake.

Total sleep time was not significantly different between HEALTH1 and FLU1 during either the Standard phase (*M* = 401 ± 85 min and *M* = 370 ± 53 min, respectively), *t*(23) = 0.92, *p* = 0.364, or during the Prophylactic phase (*M* = 413 ± 71 min and *M* = 428 ± 78 min, respectively), *t*(23) = 0.48, *p* = 0.634. Similarly, mean activity counts during wake were not significantly different between HEALTH1 and FLU1 during the Standard phase (*M* = 226 ± 37 and *M* = 231 ± 16 min, respectively), *t*(23) = 0.43, *p* = 0.665, or during the Prophylactic phase (*M* = 191 ± 45 and *M* = 156 ± 74, respectively), *t*(23) = 1.35, *p* = 0.188.

### Exercise training behavior prior to winter training

We observed significant group differences in terms of aerobic training habits, *F*(2, 45) = 3.76, *p* = 0.031. Planned comparison t- tests demonstrated that engagement in aerobic training among the FLU1 group (*M* *=* 3 ± 1 times per week) was less than the HEALTH1 group (*M* = 4 ± 1 times per week), *t*(25) = 2.32, *p* = 0.028, *d* = 0.96, but no differences were found when comparing the FLU1 group to the HEALTH2 group (*M* = 3 ± 1 times per week), *t*(30) = 0.50, *p* = 0.616, *d* = 0.19. Individuals in the HEALTH1 group engaged in aerobic training more often than the HEALTH2 group, *t*(35) = 2.27, *p* = 0.029, *d* = 0.60. In summary, healthy first-year cadets engaged more often in aerobic training than both illness-reporting first-year cadets and all second-year cadets.

Strength training was also significantly different between the three groups, *F*(2,45) = 4.08, *p* = 0.023. This effect was specific to the higher engagement in strength training among the HEALTH2 group (*M* = 4 ± 1 times per week), which was higher than both the HEALTH1 group (*M* = 3 ± 1 times per week), *t*(35) = 2.12, *p* = 0.041, *d* = 0.70 and the FLU1 group (*M* = 3 ± 1 times per week), *t*(30) = 2.69, *p* = 0.011, *d* = 1.07. No differences were observed between HEALTH1 and FLU1 groups, *t*(25) = 0.408, *p* = 0.687, *d* = 0.16.

Supplemental comparisons between HEALTH2 and all first-year cadets (i.e., independent of whether they reported illness) showed no significant difference for aerobic exercise engagement, *t*(46) = 1.24, *p* = 0.221. However, second-year cadets engaged in more strength training than combined first-year cadets, *t*(46) = 2.85, *p* = 0.006.

### Body composition

**Figure 3:**
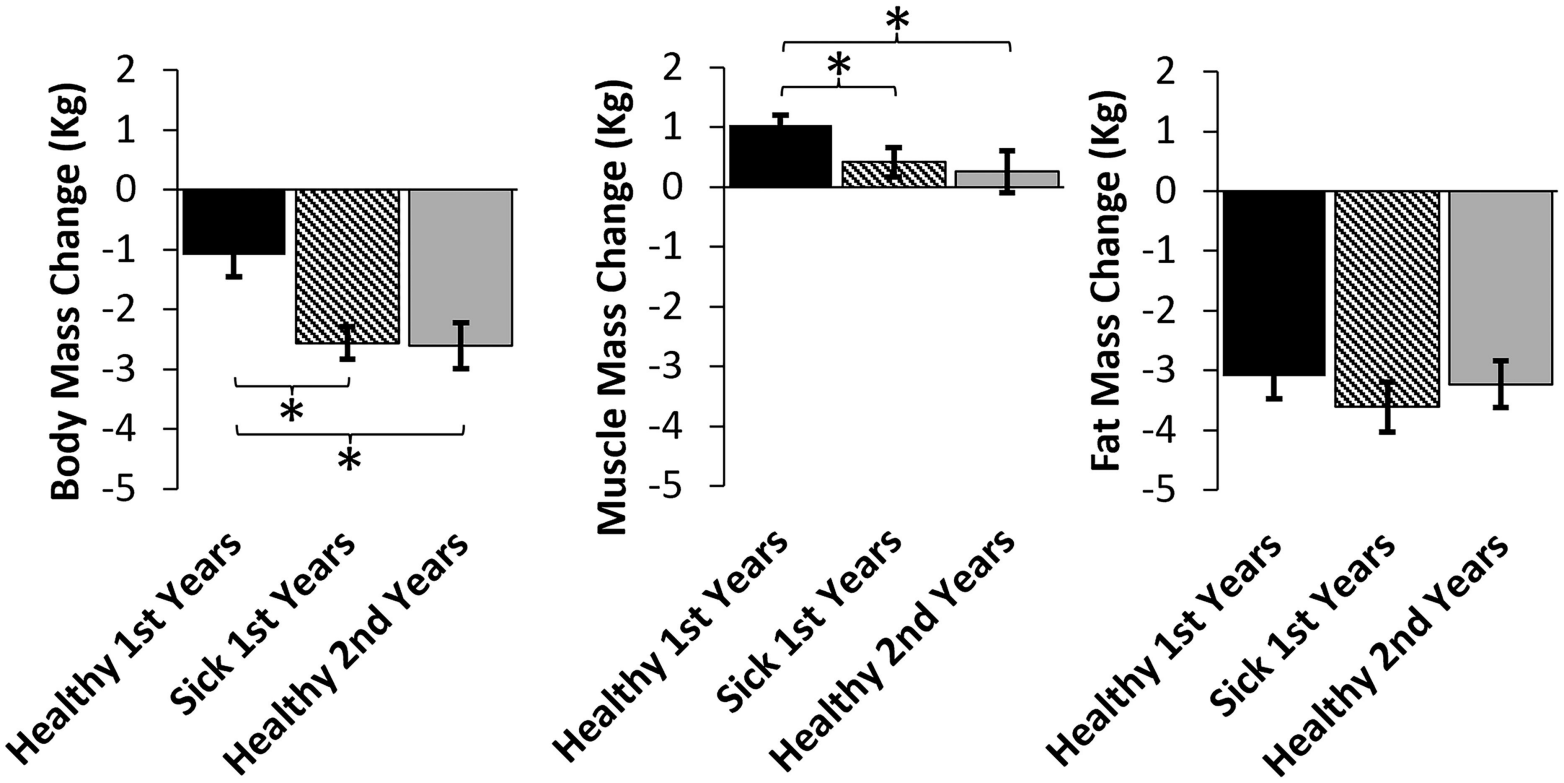
Change in body composition from before to after winter training, split by group. Overall body mass and fat mass decreased, whereas muscle mass was increased (or null). The HEALTH1 group lost significantly more body mass but gained significantly more muscle mass than the FLU1 group and the HEALTH2 group. No differences between groups were observed in the decrease in fat mass. * indicates group differences with *p* < 0.05.

Regardless of group, all cadets experienced decreased body mass, *t*(46) = −8.96, *p* < 0.001, decreased fat mass, *t*(46) = −3.27, *p* < 0.001, and increased muscle mass, *t*(46) = 0.54, *p* < 0.001, as summarized in Figure 3.

Overall body mass loss significantly differed between groups, *F* (2,44) = 5.27, *p* = 0.009. Planned comparison *t-*tests showed that body mass loss was attenuated in the HEALTH1 group (*MΔ* = 1.08 ± 1.41 kg) compared to the FLU1 group (*MΔ* = 2.56 ± 0.89 kg), *t*(24) = 3.04, *p* = 0.006, *d* = 1.25, and to the HEALTH2 group (*MΔ* = 2.60 ± 2.61 kg), *t*(34) = 2.78, *p* = 0.009, *d* = 0.95. The FLU1 and HEALTH2 groups were similar, *t*(30) = 0.08, *p* = 0.936, *d* = 0.02.

The increase in muscle mass was significantly different between groups, *F* (2,44) = 3.68, *p* = 0.033. Planned comparison *t*-tests showed that muscle mass change was greater in the HEALTH1 group (*MΔ* = 1.04 ± 0.61 kg) than both the FLU1 group (*MΔ* = 0.41 ± 0.81 kg), *t*(24) = 2.22, *p* = 0.036, *d* = 0.86, and the HEALTH2 group (*MΔ* = 0.26 ± 1.61 kg), *t*(34) = 2.61, *p* = 0.013, *d* = 0.63. No differences were observed for change in muscle mass between the FLU1 group and the HEALTH2 group, *t*(30) = 0.08, *p* = 0.936, *d* = 0.12. Finally, change in fat mass was not significantly different between groups, *F*(2,44) = 0.34, *p* = 0.713, *η_p_^2^* = 0.63.

### Mood

**Figure 4:**
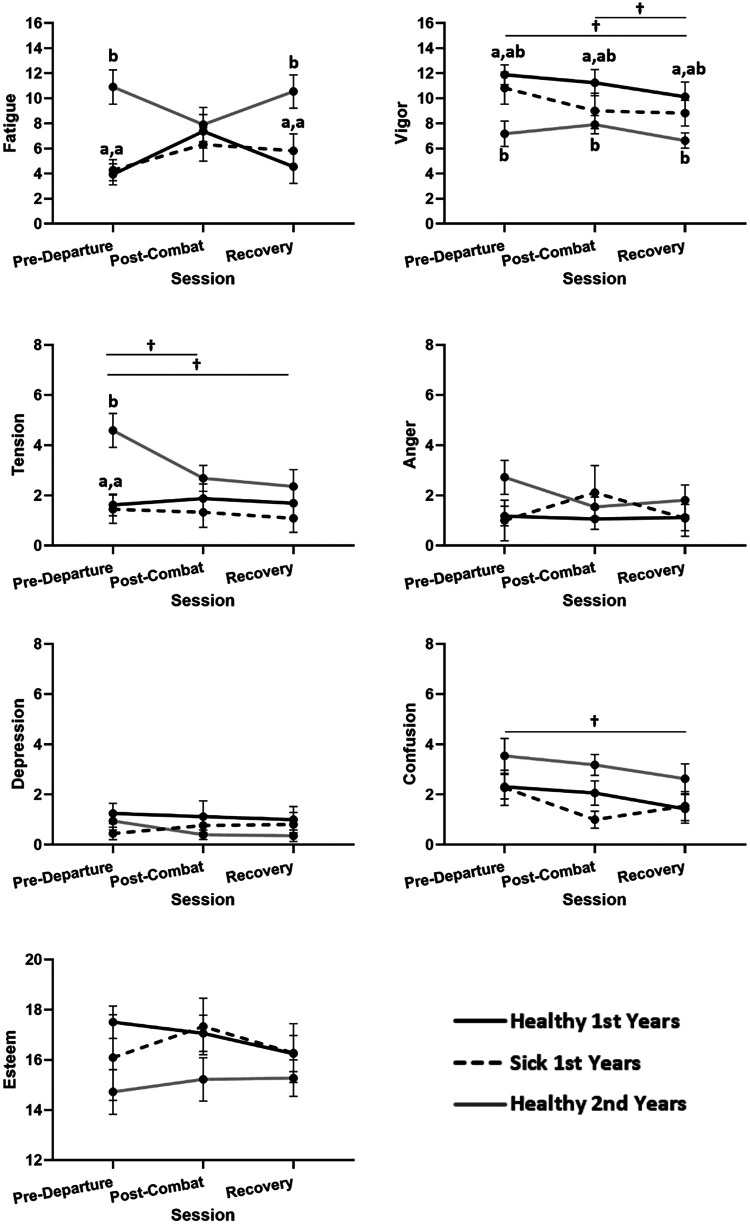
3 × 3 group by phase effects from linear mixed models for POMS subscales. The letters above the points denote significant between-group differences within each session; groups with different letters differ significantly, whereas groups sharing a letter do not differ significantly. Horizontal lines with † denote signiﬁcant session effects when collapsed across all groups. Annotations reflect significant *post hoc* differences as tested only if the omnibus tests were significant; *post hoc* comparisons for Esteem were not significant. Tension, Anger, Depression, and Confusion are log-transformed values. Error bars represent one standard error of the mean.

Changes in mood between groups and across phase are reported in Table 1 and illustrated in Figure 4. The significant results present two patterns depending on the first-year or second-year cadets' roles: Whereas the first-year cadets who participated in combat (HEALTH1) demonstrated increased fatigue immediately following the Combat phase—thus reflecting their physical demands—second-year cadets (HEALTH2) had the poorest mood during the pre-departure (i.e., high fatigue, high tension, and low vigor) and end (i.e., high fatigue) stages of the winter training, during which preparatory and clean-up were required. Supplementary material reports means and standard deviations of each group POMS factor (Supplementary Table S1) and a sensitivity analysis assessing only healthy cadets (i.e., HEALTH1 and HEALTH2) through a 2 × 3 Group by Phase ANOVA (Supplementary Table S2). In summary, the results were comparable.

**Table 1:** Results from 3 × 3 group (HEALTH1, FLU1, and HEALTH2) by phase (standard, post-combat, and recovery) linear mixed model for each POMS factor.

Factor	Between F(2,50)	*p*	η_p_^2^	Within F(2,97)	*p*	η_p_^2^	Inter. F(4,97)	*p*	η_p_^2^
Fatigue	7.11	0.002*	0.22	0.55	0.579	0.01	5.45	0.001*	0.18
Tension	4.44	0.017*	0.17	3.78	0.026*	0.05	1.52	0.201	0.09
Anger	1.32	0.276	0.0.05	0.25	0.773	<0.001	1.12	0.348	0.04
Vigor	9.86	<0.001*	0.28	4.32	0.016*	0.08	0.74	0.562	0.03
Depression	0.96	0.389	0.04	0.232	0.794	<0.001	0.760	0.554	0.03
Confusion	2.95	0.062	0.12	4.176	0.018*	0.07	1.665	0.165	0.03
Esteem	3.45	0.039*	0.12	0.340	0.713	<0.001	0.420	0.794	0.02

*n.b.*, Denominator degrees of freedom are rounded from Satterthwaite's approximations to the nearest whole integer. See Supplementary Table S3 for precise degrees of freedom values.

### Sleepiness

Self-reported sleepiness was higher in the FLU1 group (*M* = 6.83 ± 0.98) than the HEALTH1 group (*M* = 5.41 ± 1.93) during the 'Standard' phase, thus those who experienced illness felt sleepier even prior to the first onset of illness, *t*(21) = 2.31, *p* = 0.032, *d* = 0.91.

### Perceived stress

**Figure 5:**
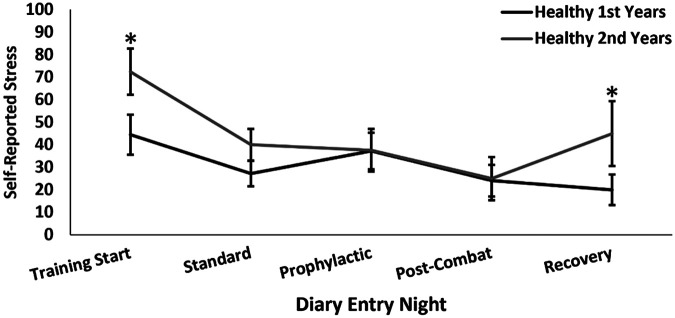
Elevated stress levels among healthy first-year cadets and healthy second-year cadets, none of whom reported flu/illness symptoms. Sufficient data were collected for five diary entries, at the start of training, and one at each phase (Standard, Prophylactic, Post-Combat, and Recovery). The second-year cadets (i.e., the group with an operational command-and-control role) reported significantly higher levels of stress than the first-year cadets (i.e., the group with more physical demands) at the training's start and at the Recovery phase, thus suggesting that they were not psychologically prepared for the duties associated with preparation or clean-up phases. Error bars represent one standard error. * indicates group differences at that phase significant at *p* < 0.05.

No group differences were observed on subjective daily stress levels, *F*(1,32.03) = 0.66, *p* = 0.42, *η_p_^2^* = 0.02; however, we observed significant within-subjects effects, *F*(4,90.98) = 8.30, *p* < 0.001, *η_p_^2^* = 0.27 and a group by phase interaction, *F*(1, 90.98) = 3.80, *p* = 0.006, *η_p_^2^* = 0.14. These effects were driven by elevated levels of stress at the start and at the end of the training for the second-year cadets only, as illustrated in Figure 5.

### Likelihood of reporting illness

Older age was predicted to increase susceptibility to developing illness (*β* = 0.42; *λ* = 1.52), and higher frequency of aerobic exercise was predicted to decrease susceptibility to developing illness (*β* = −0.97; *λ* = 0.37). Adding trait resilience and strength training as predictors did not, however, improve the model's goodness-of-fit significantly. AIC difference values suggest that the additional predictors in the model improved the expected predictability (see Table 2).

**Table 2:** Cox survival analysis for each model using forward variable selection.

Test model (Predictors)	Log-likelihood	AIC difference	Comparison model	*p*	Decision
M1 (Age)	−30.59	6.04	Null	0.004	Keep
M2 (Age + Resilience)	−26.56	−1.84	M1	0.690	Reject
M3 (Age + Aerobic)	−22.39	6.34	M1	0.003	Keep
M4 (Age + Aerobic + Anaerobic)	−21.51	−0.23	M3	0.183	Reject

Age, trait resilience, aerobic fitness habits, and anaerobic fitness habits were hypothesized predictors of experiencing flu-like symptoms during training. Listed *p*-values reflect likelihood ratio tests between test and comparison models. M, model; AIC, akaike information criterion.

## Discussion

The goal of the present study was to assess known performance factors before, during, and after a winter training in volunteer cadets. The occurrence and spread of a flu-like viral infection during the training created a naturalistic experiment, which we used to investigate differences in the vulnerability to illness, thereby dividing our participants into three groups: ill first-year cadets, healthy first-year cadets, and healthy second-year cadets (none of the second-year cadets reported any symptoms, nor were they granted medical leave).

Theoretical models on maintaining soldier readiness (10–13) have observed that soldier performance varies according to non-modifiable demands of the mission and from modifiable factors. We observed that older age (i.e., a non-modifiable factor) and less frequent aerobic exercise training habits (i.e., a modifiable factor) are significant predictors of susceptibility to developing illnesses. Loss of fat mass after training was observed in all groups. Regarding psychological variables, response to training was manifested through high levels of subjective sleepiness, subjective stress, and poor mood (i.e., modifications of fatigue, vigor, and tension), which all differed by group and by phases of training.

Flu-like illnesses are common in military contexts (28, 29, 32). Though incidental, our findings of the viral infection are not surprising in a military setting. Indeed, the demands of the winter training comprised sleep restriction and continuous physical exercise, both of which have long been known to decrease immune function (28). Sleeping less than 6 h per night is associated with a higher susceptibility to respiratory infection in both civilians (39) and soldiers during military training, resulting in lost training days (29). Also, acute respiratory illness and influenza are more prevalent during the winter (40), and soldiers sleeping in barracks report the fewest influenza outbreaks (41)—a similar finding to our results, showing that only close-quarter tent-sleepers (i.e., first-year cadets) but not barracks sleepers (i.e., second-year cadets) reported illness.

Additionally, the heavier physical demands on first-year cadets compared to second-year cadets might explain the high prevalence of illness in first-year cadets only. Sports science has linked physical load and the occurrence of infection, mainly describing infections as symptoms of overtraining syndrome (42). Evidence of immunological changes manifested as increased susceptibility to infectious illnesses, such as upper respiratory tract infections, can show a viral etiology following excessive exercise (42–44).

Military culture regards junior soldiers as being less able to endure due to a lack of experience. This perception was refuted in the 19th-century work by Thomson (45), who demonstrated that British soldiers with more campaign experience were more likely to experience disease-related fatality. Recent findings have also shown that Drill Sergeants are more likely to report depression or anxiety if they have more experience (46). Within our sample of first-year cadets, those who experienced illness were marginally older and had significantly more months of military experience, again challenging the assumption of seasoned veterans as more resilient.

Nevertheless, the practical significance of these differences should be considered; indeed, despite statistical differences and strong effect sizes as indicated by Cohen (47), the age range of these cadets was narrow (i.e., 20–27 years). We hypothesize that the main variable of interest here would be previous military experience rather than age. Rather than a physiological or metabolic effect of age (which is unlikely considering the narrow range and limited difference), the effect of experience seems more likely. Further research replicating these findings in a similar cohort with more variable levels of experience can boost the practical significance of the present findings.

First-year cadets who did not report symptoms engaged in aerobic exercise on average 1.25 times more per week than those who reported symptoms. Moreover, survival analysis indicated that, alongside older age, less engagement in aerobic training was a significant predictor of susceptibility to illness. Despite the fact that we assessed self-reported exercise training and not aerobic fitness, our findings indirectly support the ample research on the importance of aerobic fitness for preventing performance decrement (14, 15, 17, 18). These findings are important from a mitigation standpoint: Whereas age and military experience are not modifiable factors, aerobic fitness can be improved by engagement in aerobic training.

The changes in body composition reflect the physiological toll of the training and confirm numerous similar previous reports (48–51). All cadets, regardless of group, lost body mass, which was largely driven by the loss of fat mass. These results reflect a negative energy balance (more energy expenditure than energy intake). Although nutritional/energy intake was not recorded in the present study, due to operational constraints preventing this from being adequately recorded, previous studies have shown high energy expenditure during military field training (52) and negative energy balance in a cold environment (51). The loss of fat mass in a cold temperature context might also partly reflect burning fat for warmth retention, as brown adipose tissue burns to retain warmth (53).

Trait resilience was not significantly different between groups and was not a predictor of likelihood of developing flu/illness. However, the methodological debate around measuring resilience, hardiness, and mental toughness might explain our null finding (54).

Even prior to the first report of illness, cadets who eventually did get sick reported more sleepiness than those who did not. Given that sleep supports immune function (55), it is possible that sleepiness was an immune response to the illness before it was fully realized. Specifically, secretion of cytokines following inflammation is widely accepted to induce sleepiness, possibly as an adaptive response for energy conservation (56–58). Alternatively, these results might be explained through the difference in aerobic fitness between cadets, i.e., those who did not experience symptoms might have a higher effort tolerance (possibly through aerobic fitness; see above), thus they were less likely to report feeling sleepy during the Standard phase.

Perceived stress was higher at the beginning and end of the winter training for second-year cadets, specifically at the beginning of training (i.e., prior to the Standard phase) and during the Recovery phase. A similar pattern emerged among POMS factors: Fatigue was greatest at the Standard and Recovery phases among second-year cadets, during which fatigue scores were lowest among first-year cadets. Similarly, although tension was consistently low in first-year cadets, second-year cadets reported high tension during the Standard phase. Through interviews with soldiers, Doody et al. (59) showed that during pre-deployment, the increased administrative tasks and workload are perceived as stressors. Our findings suggest that, whereas military operations are inevitably stressful, the strategic planning and wrapping-up aspects can induce psychological fatigue—especially for soldiers with more command-and-control responsibility—thus these phases should not be overlooked in these specific groups.

### General discussion and future directions

We recently proposed a model outlining several factors that accompany military deployment. We showed that often environmental and work constraints can compromise both individual health and the mission outcome (11). The occurrence of a viral illness in our study sample presented us with a naturalistic experiment to verify some of the potential predictors for disease occurrence in a typical military deployment context (high physical and/or mental load under military communal living conditions). In the present study, we acknowledge that some effects, for example, sleep restriction, are unalterable constraints of the mission, despite its known detriments. However, engaging in aerobic training (i.e., prior to the mission) is a modifiable component that can attenuate performance deterioration and improve mission success, possibly via increased aerobic fitness and therefore more capability to tolerate multistressor environments. Additionally, we found that, once training has already started, recovery proves challenging, as indicated by the limited sleep opportunity, and it is thus important to prepare optimally. Soldiers and tactical athletes might be mentally preparing for the mission itself, but the unexpected practical challenges that arise in preparation for, or following the mission, are also taxing.

### Limitations

We designed hypotheses according to the existing winter training schedule, and no control group was available for this study. In this way, the study was more observational than experimental, despite our ability to assess physiological and psychological differences between two types of cadets attending the training (i.e., first-year and second-year cadets with different roles and schedules). In addition, we made use of the incidental reports of illness symptoms, at the expense of sample sizes, which were also different between groups.

Regarding the methodology of assessment, a complete picture of the performance triad or the pillars of health would have required much more detailed measurements. As an example, nutrition and hydration assessment would demand an objective measure of both the quality and quantity of food intake on the one hand, and doubly labeled water measurements on the other hand. However, due to operational constraints and access to cadets, we used the proxy measurement of body composition.

Furthermore, operational constraints limit our statistical power to observe additional group differences; therefore, this study is observational by nature of its sample size, incidental flu reports, and real-world study design. Whereas sufficient findings with strong effect sizes were nevertheless observed in some cases, this study might have been underpowered to detect other between-group differences.

Alternatively, in the case of null group differences in trait resilience, the Connor-Davidson Resilience scale might not have been the most adequate in the current population. Other instruments, such as the Hardiness scale (60) might have identified Challenge, Commitment, or Acceptance subfactors to observe differences between groups. Furthermore, several researchers have developed instruments specifically targeting military populations (61, 62), which would have been a more appropriate methodological choice.

Our findings observe psychological and physiological outcomes of tactical athletes during Arctic military training. Future studies should consider the use of objective operational measurements of performance during the training itself (e.g., ski marches and live fire exercises) to evaluate the operational cost of the current findings.

## Conclusion

Intensive military training periods comprise threats to performance, most of which are inherent to the activity and necessary to reach the training objectives. Prior aerobic training habits are a strong indicator of maintaining physical readiness during missions, and both age and aerobic training are the strongest predictors of resisting flu/illness. Physically, this winter training decreased fat mass, indicating a relative energy deficit. Psychologically, fatigue is somewhat increased during physical demands of training; soldiers with strategic demands should be aware that stress and mood can be impaired at all phases of a mission, including preparatory and clean-up phases. This study showcases the several physiological and psychological effects that impede a soldier's readiness.

## Data Availability

The datasets presented in this article are not readily available because military data is confidential and is not available for public viewing/sharing. Requests to access the datasets should be directed to nicholas.vandenberg@unb.ca.
