# Supplementary material for: Physical and mental load of tactical athletes during Arctic military training
Source: Front Sports Act Living. 2026 Jul 14;8:1861009. doi: 10.3389/fspor.2026.1861009 (PMC13410677; doi:10.3389/fspor.2026.1861009)
Supplement: Supplementary file 1 [file Supplementaryfile1.docx]

Supplemental Material for van den Berg et al. (2026) Physical and mental load of tactical athletes during arctic military training, *Frontiers in Sports and Active Living.*

Table S1. Means and Standard Deviations for each of the POMS factors, at each timepoint.

| POMS Factor | Standard | | Post-Combat | | Recovery | |
| --- | --- | --- | --- | --- | --- | --- |
| *Group* |  |  |  |  |  |  |
|  | M | SD | M | SD | M | SD |
| Fatigue |  |  |  |  |  |  |
| *HEALTH1* | 3.93 | 2.88 | 7.37 | 4.36 | 4.56 | 3.63 |
| *FLU1* | 4.27 | 2.79 | 6.33 | 4.00 | 5.81 | 4.44 |
| *HEALTH2* | 10.91 | 6.41 | 7.91 | 6.36 | 10.54 | 6.23 |
| Tension^†^ |  |  |  |  |  |  |
| *HEALTH1* | 1.62 | 1.70 | 1.87 | 2.30 | 1.68 | 2.46 |
| *FLU1* | 1.45 | 1.86 | 1.33 | 1.80 | 1.09 | 1.86 |
| *HEALTH2* | 4.59 | 3.15 | 2.68 | 2.41 | 2.36 | 3.11 |
| Anger^†^ |  |  |  |  |  |  |
| *HEALTH1* | 1.18 | 1.55 | 1.06 | 1.65 | 11.12 | 2.09 |
| *FLU1* | 1.00 | 2.68 | 2.11 | 3.58 | 1.09 | 2.38 |
| *HEALTH2* | 2.72 | 3.18 | 1.54 | 1.87 | 1.81 | 2.83 |
| Vigour |  |  |  |  |  |  |
| *HEALTH1* | 11.87 | 3.18 | 11.25 | 4.18 | 10.12 | 4.66 |
| *FLU1* | 10.81 | 4.26 | 9.00 | 4.21 | 8.81 | 3.40 |
| *HEALTH2* | 7.18 | 4.72 | 7.90 | 3.40 | 5.63 | 2.82 |
| Depression^†^ |  |  |  |  |  |  |
| *HEALTH1* | 1.25 | 1.61 | 1.12 | 2.52 | 1.00 | 2.09 |
| *FLU1* | 0.45 | 0.82 | 0.77 | 1.39 | 0.81 | 1.60 |
| *HEALTH2* | 0.95 | 1.70 | 0.40 | 0.90 | 0.36 | 1.09 |
| Confusion^†^ |  |  |  |  |  |  |
| *HEALTH1* | 2.31 | 1.95 | 2.06 | 1.94 | 1.43 | 2.27 |
| *FLU1* | 2.27 | 2.32 | 1.00 | 1.11 | 1.54 | 1.91 |
| *HEALTH2* | 3.54 | 3.27 | 3.18 | 1.96 | 2.63 | 2.78 |
| Esteem |  |  |  |  |  |  |
| *HEALTH1* | 17.5 | 2.58 | 17.06 | 2.88 | 16.25 | 2.88 |
| *FLU1* | 16.09 | 5.66 | 17.33 | 3.74 | 16.27 | 3.90 |
| *HEALTH2* | 14.72 | 4.18 | 15.22 | 4.04 | 15.27 | 3.41 |

^†^ Indicates log-transformed values

Table S2. Results for each of the POMS subscales after excluding the FLU1 group, thus examining only healthy cadets via a 2x3 Group (HEALTH1, HEALTH2) by Phase (Standard, Post-Combat, Recovery) ANOVA for between-groups, within-subjects, and interaction effects.

|  | ***Between***  *F(1,36)* | *p* | *η_p_^2^* | ***Within***  *F(2,72)* | *p* | *η_p_^2^* | ***Inter.***  *F(2,72)* | *p* | *η_p_^2^* |
| --- | --- | --- | --- | --- | --- | --- | --- | --- | --- |
| Fatigue | 9.01 | <0.001 | 0.200 | 0.03 | 0.965 | 0.001 | 8.93 | <0.001 | 0.199 |
| Tension | 5.01 | 0.031 | 0.122 | 4.50 | 0.071 | 0.111 | 3.53 | 0.034 | 0.089 |
| Anger | 2.45 | 0.126 | 0.064 | 1.16 | 0.231 | 0.031 | 0.79 | 0.458 | 0.021 |
| Vigour | 17.11 | <0.001 | 0.322 | 4.12 | 0.020 | 0.103 | 4.92 | 0.559 | 0.120 |
| Depression | 1.51 | 0.227 | 0.040 | 1.25 | 0.290 | 0.034 | 0.318 | 0.729 | 0.009 |
| Confusion | 3.18 | 0.083 | 0.081 | 2.47 | 0.092 | 0.064 | 0.010 | 0.986 | 0.000 |
| Esteem | 6.40 | 0.016 | 0.151 | 0.158 | 0.854 | 0.004 | 0.703 | 0.498 | 0.019 |

Table S3. Precise values of denominator degrees of freedom when conducting the 3x3 Group by Phase linear mixed models for 7 POMS subfactors, following Satterthwaite’s degrees of freedom approximations correction for unequal groups. Results are displayed in Table 1.

|  | ***Between*** | ***Within*** | ***Interaction*** |
| --- | --- | --- | --- |
| Fatigue | 49.49 | 96.93 | 96.77 |
| Tension | 49.83 | 97.44 | 97.23 |
| Anger | 49.91 | 97.70 | 97.45 |
| Vigour | 49.93 | 97.58 | 97.37 |
| Depression | 49.82 | 97.41 | 97.21 |
| Confusion | 48.98 | 96.45 | 96.28 |
| Esteem | 50.34 | 98.62 | 98.28 |
